# Blood manganese and non-alcoholic fatty liver disease in a high manganese exposure area in China

**DOI:** 10.1186/s41043-023-00467-2

**Published:** 2023-11-06

**Authors:** Liping Wu, Yanqi Lan, Ze Yu, Yanhong Wang, Wei Liao, Guoqiang Zhang, Li Wang

**Affiliations:** 1 Department of Hepatobiliary Surgery, Zhoushan Hospital, Wenzhou Medical University, Zhoushan, 316021 China; 2 Department of Epidemiology and Biostatistics, Institute of Basic Medical Sciences Chinese Academy of Medical Sciences, School of Basic Medicine Peking, Union Medical College, 5 Dong Dan San Tiao, Beijing, 100730 China

**Keywords:** NAFLD, Manganese, Case–control study, High manganese exposure area

## Abstract

**Background and aims:**

Manganese (Mn) deficiency and intoxication may affect nonalcoholic fatty liver disease (NAFLD) risk differently. We aimed to explore the association between blood Mn and NAFLD in an area with high Mn exposure in drinking water.

**Methods:**

We conducted a case–control study among 1407 patients with NAFLD and 1774 sex- and age-matched healthy controls in a physical examination population in Zhoushan hospital, Zhejiang province in China. We used the restricted cubic splines method to investigate the dose–response relationship. Logistic regression models were applied to determine the risk of NAFLD, and severity of NAFLD.

**Results:**

The blood Mn concentration was higher in the NAFLD group than in the control group in women (16.1 ± 6.2 μg/L vs. 14.7 ± 6.4 μg/L, P = 0.022) and men (14.5 ± 6.3 μg/L vs. 13.6 ± 6.8 μg/L, P < 0.001). We found an inverted L shape relationship between blood Mn and NAFLD in both women and men. Compared to the lowest quartile, the adjusted odds ratio (OR) and 95% confidence interval (CI) of NAFLD for the highest quartile group was 1.646(1.222,2.217), 1.494(1.082,2.061), and 3.146(1.285,7.701) for the total population, men, and women. The positive relationship was only observed in those with fibrosis-4 score < 1.30 and normal alanine transaminase. Stratified analysis showed an interaction between smoking (P = 0.073), alcohol drinking (P = 0.013), and Mn, with a more prominent effect on the NAFLD in the never-smokers (OR = 2.153, 95% CI 1.408–3.290) and drinkers (OR = 2.596, 95% CI 1.608–4.191).

**Conclusion:**

Higher blood Mn is associated with an elevated NAFLD risk in the high Mn exposure areas, especially in nonsmokers and drinkers. Further studies are needed to verify this result in the areas with high Mn exposure.

## Introduction

Non-alcoholic fatty liver disease (NAFLD) has become the most common liver disease in the world, including China. It is a disease with excessive accumulation of hepatic fat after excluding the secondary causes of fatty liver, accounting for one-fourth of adults in the general population [1]. NAFLD can lead to increased risks of intrahepatic and extrahepatic adverse outcomes, including cancers, cardiovascular diseases, and chronic kidney disease [2–4], and bring enormous clinical and economic burden to individuals and society [5]. However, there are currently no approved medications for NAFLD, and lifestyle changes aimed at weight loss, such as diet control and physical exercise, are the main recommended interventions [6]. Therefore, exploring the risk factors of NAFLD, especially the modifiable risk factors of NAFLD, and carrying out targeted interventions will have important public health significance for reducing the disease burden of NAFLD.

As an essential element in the human body, Manganese (Mn) metals play a crucial role in regulating carbohydrate and lipid metabolism, improving immune function, as well as oxidative stress; and then end up in chronic diseases [7]. Mn enters the human body through the air, water, food, medicine, and other consumer goods. Both deficiency and intoxication of Mn are associated with metabolic and neuropsychiatric diseases, suggesting its relationship with NAFLD. In a small sample of patients with NAFLD diagnosed by liver biopsy, liver Mn content and whole blood Mn content were inversely associated with the degree of steatosis [8]. However, a positive correlation between serum Mn content and NAFLD was reported in men from southern China [9]. As a key activator of biological enzymes-mediated metabolic diseases-associated pathophysiological processes [10], Mn exposure level may be a biomarker of the development of NAFLD since NAFLD is fundamentally a disease of altered systemic metabolism. Mn is an essential nutrient but becomes toxic at high levels, which may explain the inconsistent results between blood Mn and NAFLD in different populations [8, 9].

Zhoushan City is a prefectural-level administrative area with an archipelago. The concentration of Mn in drinking water in Zhoushan City exceeds the standard [11], suggesting the difference in the association between Mn and NAFLD in the inhabitants of this city. Therefore, this study aimed to explore the association between blood Mn and NAFLD in an area with high Mn exposure.

## Materials and methods

### Study population

Zhoushan Hospital is the only Grade III Class A general hospital in Zhoushan City, Zhejiang province in China. The physical examination center of Zhoushan Hospital provides physical examination services for employees of enterprises and institutions. This study was a case–control study based on subjects who worked in enterprises and institutions in Zhoushan City and had a physical examination in this center from September 1, 2017, to December 31, 2018. We randomly selected 1407 subjects (161 females and 1246 males) who underwent abdominal ultrasonography exams and were diagnosed with NAFLD. According to the American Association for the Study of Liver Diseases Practice Guidance for the diagnosis of NAFLD [12], all the cases were diagnosed with fatty liver disease by abdominal ultrasonography scans after excluding those with (1) hepatitis B virus infection or excessive alcohol drinking (alcohol consumption of ≥ 30 g ethanol/day for men and ≥ 20 g ethanol/day for women), or missing data on alcohol consumption; or (3) with history of cancers. Then, simultaneously, age- and sex-matched controls were randomly selected in the population under the physical examination. All the controls underwent abdominal ultrasonography scans to exclude the fatty liver disease and had the same exclusion criteria as the NAFLD cases. All cases and controls had signed informed consent.

### Data collection

We conducted a questionnaire survey to collect demographic data (age, sex, education level, whether or not born in Zhoushan city, lifestyle factors (smoking and alcohol drinking status, physical activity), and medical history. Subjects who smoked at least 1 cigarette per day with continuous smoking for more than 6 months were defined as current smokers, and those who did not smoke in the past 6 months before the survey were defined as ex-smokers. Subjects with alcohol consumption > 0-g ethanol/day were defined as drinkers. According to the Guidelines for Data Processing and Analysis of the International Physical Activity Questionnaire [13], Metabolic equivalents (METs) were calculated as follows: METs (MET-minutes/week) = metabolic coefficient of physical activity × exercise frequency (day/week) × exercise time (minutes/day). The metabolic coefficients of physical activity were assigned as 8 MET for vigorous physical activity, 4 MET for moderate physical activity, and 3.3 MET for walking. Blood pressure and abdominal ultrasonography were measured. We used an auto-analyzer (Hitachi 747; Hitachi, Tokyo, Japan) to measure all the participants’ serum fasting blood glucose (FBG), total cholesterol (TC), triglyceride (TG), low-density lipoprotein cholesterol (LDL-c), high-density lipoprotein cholesterol (HDL-c), alanine transaminase (ALT), aspartate transaminase (AST), albumin (ALB), gamma-glutamyl transpeptidase (GGT), and platelets. Hyperlipidemia was defined as TG concentration ≥ 1.7 mmol/L, HDL concentration < 1.04 mmol/L, or self-reported history of hyperlipidemia [14]. Hypertension was defined as systolic blood pressure of ≥ 140 mmHg, diastolic blood pressure of ≥ 90 mmHg, or medical history of hypertension [15]. Diabetes was defined as fasting blood glucose ≥ 7.0 mmol/L or medical history of diabetes.

### Measurement of blood Mn

Fasting venous whole-blood samples were collected and stored at – 80 °C within 24 h. The method for examining the blood Mn concentration was described previously [16]. Firstly, we diluted 200 μL of blood using 0.5% nitric acid to 1.0 mL. After centrifugation at 13,000 r/min, we added 2.1 mL 0.5% nitric acid and 150 μL internal standard solution (germanium, 200 ng/mL) to the 750 μL supernatant. Then, we used inductively coupled plasma mass spectrometry (iCAPQc, Thermo Fisher) to measure the Mn concentrations. We used pooled blood samples once in every 15 samples as quality control. All the metal concentrations were within the linear range. Intra-assay and interassay coefficient of blood Mn were < 10%.

### Statistical analysis

We described and compared the characteristics of cases and controls. We used the Anderson–Darling test to test the normality of continuous variables. If following normal distribution, continuous variables were presented as mean ± standard deviation and compared by the Student’s t-test. If not, they were presented as median (quartiles) and compared by the Manne-Whitney test. Categorical variables were presented as number (%) and compared by Pearson chi-square test or Fisher exact probability test. We used restricted cubic splines with three knots to explore the dose–response relationship between Mn and NAFLD in the total population, males and females. Then, we divided all the participants into quartiles according to the control blood Mn levels. Using the lowest quartile group as the reference, we conducted unadjusted-, age-, sex-adjusted, and multivariate-adjusted (further adjusted METs, ALT, TC, GGT, LDL, TG, hypertension, diabetes, smoking, and alcohol drinking status) logistic regression to estimate the odds ratio (OR) and 95% confidence interval (CI) for NAFLD risk in the other three groups. In addition, we used a logistic regression model to test the linear trend by treating the median of each quartile as independent variables. We redefined the severity of NAFLD using the fibrosis-4 score (FIB-4), FIB-4 = Age (years) × AST [U/L]/(Platelet [10^9^/L] × (ALT [U/L])1/2) [17] and ALT, and explored the relationship between Mn and the severity of NAFLD. Furthermore, we conducted a stratified analysis to test the potential interaction between diabetes, hypertension, smoking, alcohol drinking, and Mn in NAFLD development. We used SAS 9.4 (SAS Institute, NC) for analysis and judged P < 0.05 under the two-sided test as statistical significance.

## Results

### Characteristics of the study population

In total, 161 NAFLD and 836 non-NAFLD females, and 1246 NAFLD and 938 non-NAFLD males were included in the study (Table 1). In both men and women, the NAFLD group had a higher ALT, AST, TC, TG, LDL, GGT, FBG, SBP, and DBP than the non-NAFLD (p < 0.05). Also, the NAFLD group had a higher proportion of diabetes, hyperlipidemia, and hypertension than the non-NAFLD. For smoking status, the subjects with NAFLD had a higher proportion of current smoking than the non-NAFLD in men (47.3% vs. 40.8%, P < 0.001) but not in women (17.7% vs. 21.4%, P = 0.40).

**Table 1:** Characteristics of the study population

		Female			Male	
	Non-NAFLD	NAFLD	P-value	Non-NAFLD	NAFLD	P-value
N	836	161		938	1246	
Age (years)	44.7 ± 10.9	45.1 ± 12.0	0.77	42.7 ± 10.3	42.7 ± 10.0	0.98
Born in Zhoushan	238 (82.6)	126 (80.8)	0.97	580 (63.4)	856 (69.9)	0.001
High school or above	259 (89.9)	127 (80.4)	0.005	851 (91.8)	1130 (91.4)	0.71
Smoke			0.4			< 0.001
Never smoker	44 (16.2)	32 (21.8)		440 (49.3)	489 (40.6)	
Ex-smoker	169 (62.4)	89 (60.5)		88 (9.9)	145 (12.1)	
Current smoker	58 (21.4)	26 (17.7)		364 (40.8)	569 (47.3)	
Drinkers	25 (8.6)	36 (22.4)	< 0.001	311 (33.2)	671 (53.9)	< 0.001
METs	816.8	1039.5	0.15	1039.5	1039.5	0.07
METs-min/week	(346.5–1260.0)	(346.5–1386.0)		(519.8–1386.0)	(383.3–1386.0)	
ALT (U/L)	15.0 (11.0– 19.0)	24.0 (17.0–35.0)	< 0.001	21.0 (16.0–29.0)	36.0 (26.0–53.5)	< 0.001
AST (U/L)	20.0 (18.0–24.0)	23.0 (20.0–29.5)	< 0.001	23.0 (20.0–27.0)	27.0 (23.0–34.0)	< 0.001
TC (mmol/L)	5.1 ± 1.0	5.4 ± 1.1	< 0.001	5.0 ± 0.9	5.3 ± 1.0	< 0.001
TG (mmol/L)	0.9 (0.7– 1.2)	1.6 (1.2–2.1)	< 0.001	1.3 (0.9–1.7)	2.0 (1.4–2.8)	< 0.001
HDL (mmol/L)	1.6 ± 0.3	1.3 ± 0.2	< 0.001	1.3 ± 0.3	1.2 ± 0.2	< 0.001
LDL (mmol/L)	2.7 ± 0.7	3.1 ± 0.7	< 0.001	2.8 ± 0.7	3.0 ± 0.7	< 0.001
GGT (mmol/L)	15.0 (12.0–20.0)	23.0 (18.0–39.5)	< 0.001	25.0 (19.0–37.0)	44.0 (31.0–68.0)	< 0.001
FBG (mmol/L)	5.0 ± 0.5	5.4 ± 1.4	< 0.001	5.1 ± 1.0	5.4 ± 1.3	< 0.001
SBP (mmHg)	119.4 ± 16.1	129.7 ± 19.9	< 0.001	125.9 ± 15.9	132.0 ± 15.7	< 0.001
DBP (mmHg)	71.4 ± 10.5	77.1 ± 11.6	< 0.001	77.4 ± 10.8	82.4 ± 11.6	< 0.001
Diabetes	5 (0.6)	8 (5.5)	< 0.001	42 (4.5)	108 (8.7)	< 0.001
Hyperlipidemia	20 (2.5)	29 (20.4)	< 0.001	319 (34.0)	885 (71.0)	< 0.001
Hypertension	27 (3.4)	21 (14.3)	< 0.001	710 (76.3)	734 (59.3)	< 0.001
Manganese (μg/L)	14.7 ± 6.4	16.1 ± 6.2	0.022	13.6 ± 6.8	14.5 ± 6.3	< 0.001

Data are presented as mean ± standard deviation, median (p25-p75), or n (%)

NAFLD, non-alcoholic fatty liver disease; MET, Metabolic equivalents; ALT, alanine transaminase; AST, aspartate transaminase; TC, total cholesterol; TG, triglycerides; HDL-c, high-density lipoprotein cholesterol; LDL-c, low-density lipoprotein; GGT, gamma-glutamyl transpeptidase; FBG, fasting blood glucose; SBP, systolic blood pressure; DBP, diastolic blood pressure

### Blood Mn in the participants with NAFLD and non-NAFLD

The concentration of Mn was 16.1 ± 6.2 μg/L for NAFLD and 14.7 ± 6.4 μg/L for non-NAFLD in women (P = 0.022). The same trend was observed in men, with 14.5 ± 6.3 μg/L for cases and 13.6 ± 6.8μg/L for controls (P < 0.001).

### Association between blood Mn and NAFLD

**Fig. 1:**
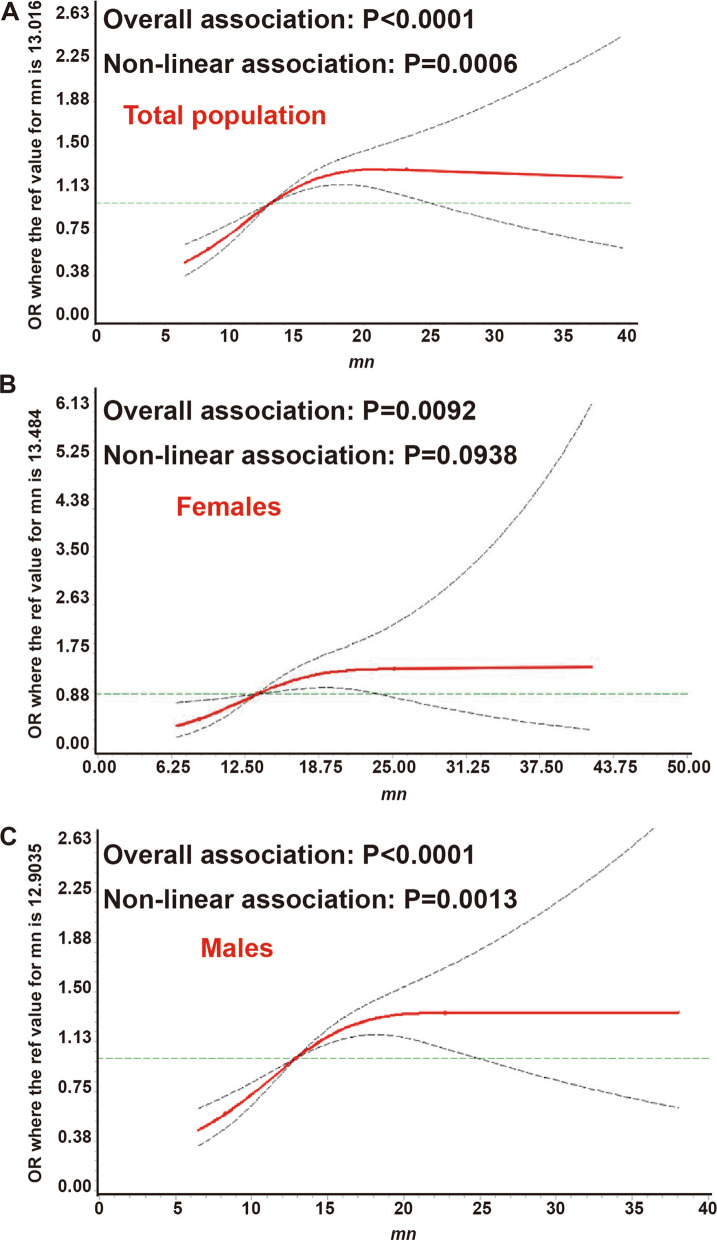
Restricted cubic spline curves for manganese and non-alcoholic fatty liver disease. **A**. Total population; **B**. Females; **C**. Males. Mn, manganese; OR, odds ratio

Restricted cubic spline curves (Fig. 1A–C) found an inverted L shape relationship between blood Mn and NAFLD in the total population (overall association P < 0.001, non-linear association P < 0.001), men (P < 0.001, non-linear association P = 0.001) and women (overall association P = 0.009, non-linear association P = 0.094). So, we further divided all the participants into four groups. Compared with the lowest quartile (Quartile 1), the NAFLD risk increased with a higher blood level of Mn in the total population, men and women in all the models (Table 2). For example, in the full-adjusted model (model 3), the OR (95% CI) for the quartile 4 group was 1.646(1.222,2.217), 1.494(1.082,2.061), and 3.146(1.285,7.701) for the total population, men, and women, respectively.

**Table 2:** Association between Mn and NAFLD

	Quartile 1	Quartile 2	Quartile 3	Quartile 4	P trend
*All*					
Cut-off (μg/L)	< 10.70	10.70–13.01	13.02–16.22	> 16.22	
Model 1	1	1.311 (1.051,1.633)	1.509 (1.211,1.88)	1.791 (1.435,2.236)	0.002
Model 2	1	1.354 (1.082,1.693)	1.552 (1.241,1.941)	1.962 (1.563,2.462)	< 0.001
Model 3	1	1.279 (0.9553,1.717)	1.346 (1.004,1.806)	1.646 (1.222,2.217)	0.045
*Females*					
Cut-off (μg/L)	< 11.04	11.04–13.47	13.48–17.69	> 17.69	
Model 1	1	1.283 (0.696,2.367)	1.282 (0.692,2.374)	2.064 (1.168,3.648)	0.002
Model 2	1	1.279 (0.691,2.365)	1.279 (0.686,2.383)	2.056 (1.162,3.638)	0.002
Model 3	1	1.767 (0.680,4.592)	2.212 (0.852,5.743)	3.146 (1.285,7.701)	0.087
*Males*					
Cut-off (μg/L)	< 10.59	10.59–12.90	12.91–16.05	> 16.05	
Model 1	1	1.363 (1.072,1.733)	1.604 (1.262,2.039)	1.918 (1.497,2.457)	0.035
Model 2	1	1.364 (1.073,1.735)	1.605 (1.262,2.041)	1.922 (1.499,2.464)	0.039
Model 3	1	1.262 (0.922,1.727)	1.313 (0.960,1.795)	1.494 (1.082,2.061)	0.19

The data are presented as odds ratio (95% confidence interval). The logistic regression models are constructed with data of the control group of those with blood Mn concentration in the lowest quartile

Model 1: univariate model;

Model 2: age-, sex-adjusted model for the total population and age-adjusted for the males and females;

Model 3: Further adjusted for metabolic equivalents, alanine transaminase, total cholesterol, triglycerides, low-density lipoprotein, gamma-glutamyl transpeptidase, hypertension, diabetes, smoking, and drinking status

### Association between blood Mn and NAFLD with different severity

Next, we used FIB-4 and ALT to reflect the severity of NAFLD (Table 3). After adjusting the covariates, the positive association between Mn and NAFLD was only observed in those with FIB-4 < 1.3. The results were consistent in the total population, men, and women, with OR (95%CI) for the quartile 4 was 1.795 (1.323,2.435), 1.559 (1.126,2.160), 3.816 (1.567,9.294), respectively. A similar phenomenon was observed in ALT. All the positive relationships were found in the NAFLD with normal ALT. The OR (95% CI) was 1.717 (1.259,2.343), 1.493 (1.067,2.088), and 3.906 (1.635,9.333) in the total population, men and women, respectively.

**Table 3:** Association between Mn and NAFLD with different severity

		Quartile 1	Quartile 2	Quartile 3	Quartile 4
All	Fibrosis-4				
	Score				
	< 1.30 (n = 1004)	1	1.406 (1.038,1.906)	1.417 (1.047,1.919)	1.795 (1.323,2.435)
	≥ 1.30 (n = 134)	1	0.818 (0.467,1.430)	1.069 (0.617,1.849)	1.032 (0.581,1.835)
	ALT (U/L)				
	< 40 (n = 677)	1	1.320 (0.969,1.798)	1.355 (0.995,1.844)	1.717 (1.259,2.343)
	≥ 40 (n = 461)	1	1.090 (0.658,1.805)	1.211 (0.735,1.994)	1.488 (0.909,2.436)
Female	Fibrosis-4				
	Score				
	< 1.30 (n = 113)	1	1.955 (0.783,4.880)	2.114 (0.842,5.303)	3.816 (1.567,9.294)
	≥ 1.30 (n = 13)	1	1.739 (0.282,10.748)	3.884 (0.440,34.307)	5.807 (0.919,36.697)
	ALT (U/L)				
	< 40 (n = 99)	1	1.838 (0.757,4.463)	2.121 (0.869,5.173)	3.906 (1.635,9.333)
	≥ 40 (n = 27)	–	–	–	–
Male	Fibrosis-4				
	Score				
	< 1.30 (n = 891)	1	1.278 (0.922,1.772)	1.469 (1.059,2.037)	1.559 (1.126,2.160)
	≥ 1.30 (n = 121)	1	0.773 (0.425,1.405)	1.246 (0.696,2.229)	0.818 (0.441,1.518)
	ALT (U/L)				
	< 40 (n = 578)	1	1.241 (0.888,1.734)	1.442 (1.031,2.019)	1.493 (1.067,2.088)
	≥ 40 (n = 434)	1	1.022 (0.609,1.717)	1.270 (0.760,2.121)	1.286 (0.780,2.121)

The data are presented as odds ratio (95% confidence interval). The multinomial logistic regression models are constructed with data of the control group of non-NAFLD subjects and unexposed group of those with blood Mn concentration in the lowest quartile

The model was adjusted for age, sex (only for the total population), metabolic equivalents, alanine transaminase, total cholesterol, triglycerides, low-density lipoprotein, gamma-glutamyl- transpeptidase, hypertension, diabetes, smoking, and drinking status

NAFLD, non-alcoholic fatty liver disease; Mn, manganese

### Stratified analysis of the association between blood Mn and NAFLD

Table 4 showed the stratified analysis of the relationship between Mn and NAFLD. There was a marginally significant or significant interaction between smoking (P = 0.073), alcohol drinking (P = 0.013), and Mn. Mn has a more prominent effect on NAFLD in the never-smokers (OR = 2.153, 95% CI 1.408–3.290) than in the ever-smokers (OR = 1.140, 95% CI: 0.737–1.762). On the contrary, the positive relationship was only observed in the drinkers (OR = 2.596, 95% CI 1.608–4.191).

**Table 4:** Stratified analysis of the association between Mn and NAFLD

	Quartile 1	Quartile 2	Quartile 3	Quartile 4	P interaction
Smoking					
Never	1	1.523 (0.992,2.338)	1.628 (1.067,2.483)	2.153 (1.408,3.290)	0.073
Ever	1	1.054 (0.692,1.605)	1.065 (0.696,1.631)	1.140 (0.737,1.762)	
Drinking					
No	1	0.996 (0.686,1.445)	0.914 (0.623,1.342)	1.236 (0.842,1.814)	0.013
Yes	1	1.979 (1.217,3.220)	2.365 (1.473,3.795)	2.596 (1.608,4.191)	
Hypertension					
No	1	1.307 (0.916,1.864)	1.335 (0.936,1.904)	1.739 (1.212,2.493)	0.66
Yes	1	1.301 (0.753,2.247)	1.515 (0.877,2.617)	1.516 (0.879,2.616)	
Diabetes					
No	1	1.386 (1.023,1.878)	1.360 (1.003,1.843)	1.733 (1.277,2.352)	0.63
Yes	1	0.243 (0.058,1.020)	0.987 (0.236,4.125)	0.796 (0.148,4.275)	

The data are presented as odds ratio (95% confidence interval). The logistic regression models are constructed with data of the control group of those with blood Mn concentration in the lowest quartile

The model was adjusted for age, sex (only for the total population), metabolic equivalents, alanine transaminase, total cholesterol, triglycerides, low-density lipoprotein, gamma-glutamyl- transpeptidase, hypertension, diabetes, smoking, and drinking status

NAFLD, non-alcoholic fatty liver disease; Mn, manganese

## Discussion

Using a case–control study in Zhoushan City (a high-water Mn exposure area), our study found a positive relationship between blood Mn and NAFLD. The conclusion was consistent in the total population, men and women. However, after defining NAFLD severity by FIB-4 and ALT, the positive was only observed in those with FIB-4 < 1.30 and normal ALT. In addition, the association between Mn and NAFLD might be modified by smoking and alcohol drinking status.

We observed an increased risk of NAFLD in the subject with higher Mn, consistent with a hospital-based cross-sectional study in the Southern area of China [9]. However, our former cohort-based case–control study in the Northern region of China found the opposite conclusion. Our study reported a negative relationship in males [18]. A different source of the study population may contribute to the heterogeneity of the results. Depending on its concentration level, Mn could be an essential trace element in the human body or a toxic heavy metal element. Mn has been demonstrated to be a nutrient that shows a typical nonmonotonic dose–response relationship with many chronic diseases, with too low or too high causing toxicity.

In the areas with normal or deficient Mn exposure, a lower level of Mn could decrease the function of Mn-superoxide dismutase, alter lipid and glucose metabolism, and result in metabolic diseases, including NAFLD. However, just as in regions like Zhoushan City with a high concentration of Mn in water, an increased Mn level may disrupt normal mitochondrial function by increasing ROS, inhibiting ATP production, and changing membrane permeability [19]. Furthermore, mitochondrial disorder can contribute to oxidative stress and increase NAFLD risk [7]. Although inconsistent, several studies reported an elevated blood Mn level in diabetes [20, 21]. In addition, a case–control study in a Chinese population found a U-shaped relationship between plasma Mn and newly diagnosed diabetes, further supporting our hypothesis [22]. That is, a positive relationship exists between Mn and NAFLD in the population living in the high-level Mn exposure areas.

Considering the severity of NAFLD according to FIB-4 and ALT levels, the positive association between Mn and NAFLD was only observed in those with FIB-4 < 1.3 and those with normal ALT. The small sample size may explain the non-significant relationship in the subjects with FIB-4 < 1.3 or those with normal ALT. Since smoking, alcohol drinking, and metabolic disorders are the risk factors for NAFLD, we also explored the interaction between their interaction between Mn. A prominent hazard effect was observed in the never smokers, with an OR(95% CI) of 2.153(1.408,3.290). No significant association between higher Mn and NAFLD in the ever smokers could be explained by shared risk factors for NAFLD, such as oxidative stress [23–25], inflammation [26, 27], and insulin resistance [28–30]. Although inconsistent [31–33], a cohort study involving self-reporting smoking status, pack-years, and urinary cotinine level has shown positive relationships between smoking and NAFLD [34]. Animal studies have also supported that smoking could exacerbate NAFLD in obese rats [35].

Contrary to smoking, a greater NAFLD risk was observed in drinkers than in non-drinkers, with an OR (95% CI) of 2.596(1.608,4.191) vs. 1.236(0.842,1.814). Consistent with our study, a cross-sectional study in China showed a positive interaction between alcohol drinking and occupational Mn exposure. A higher concentration of liver enzymes, including ALT, AST, and direct bilirubin, was observed in the drinkers than in the non-drinkers among ferro-Mn refinery company workers [36], which could explain a positive interaction of alcohol drinking and blood Mn in our population. Furthermore, animal studies showed that alcohol exposure could increase the accumulation of Mn and accelerate neurotoxicity in mice, indirectly supporting our findings [37].

Our study has several limitations. Firstly, NAFLD was diagnosed by ultrasonography but not liver histology due to the invasiveness of the latter, leading to the misclassification of NAFLD as non-NAFLD when the liver fat of < 20% [38] and underestimating the effect of Mn and NAFLD. Secondly, we used prevalent NAFLD cases but not incident cases, which may have prevalence incidence bias. However, our former study showed that there was no significant difference in blood Mn between the incident and prevalent cases [18]. Thirdly, we did not collect dietary Mn exposure in this study, which may bring about potential residual confounding. However, we did not find a significant relationship between dietary Mn and NAFLD in the former study [18], indicating not adjusting this factor would not influence the conclusion. Fourthly, we did not measure body mass index and did not adjust obesity, which may overestimate the effect of blood Mn and NAFLD. Fifthly, our former study in North China showed that exposure to metal mixtures is associated with a higher risk for NAFLD than single metal. Interactions between metals suggest the importance of balancing the various metals for health benefits [39]. Further studies should be conducted to explore the interactions between blood Mn and other metals for the occurrence of NAFLD in Zhoushan City.

In conclusion, higher blood Mn is associated with an elevated NAFLD risk in the high Mn exposure areas, especially in nonsmokers and drinkers. We call for further study, especially cohort study, to verify the relationship between Mn and NAFLD in the areas with high Mn exposure.

## Data Availability

All data generated or analyzed during this study are included in this published article. All data that obtained and analyzed during our study are available from corresponding author once reasonably requested.

## Abbreviation

NAFLD: Nonalcoholic fatty liver disease
Mn: Manganese
FBG: Fasting blood glucose
TC: Total cholesterol
TG: Triglycerides
LDL-c: Low-density lipoprotein cholesterol
HDLc: High-density lipoprotein cholesterol
ALT: Alanine transaminase
ALB: Albumin
AST: Aspartate transaminase
GGT: Gamma-glutamyl transpeptidase
OR: Odds ratio
95% CI: 95% Confidence interval
FIB-4: Fibrosis-4 score
